# Homing of Cultured Endothelial Progenitor Cells and Their Effect on Traumatic Brain Injury in Rat Model

**DOI:** 10.1038/s41598-017-04153-2

**Published:** 2017-06-23

**Authors:** Xin-bin Guo, Xin Deng, Ying Wei

**Affiliations:** Department of Neuro-interventional Radiology, The First Affiliated Hospital of Zhengzhou University, 1 Jianshe Road, Zhengzhou, 450052 China

## Abstract

Transplanted endothelial progenitor cells (EPCs) may play an important role in reestablishing the endothelial integrity of the vessels after brain injury, and contribute to neurogenesis. We, therefore, tested the homing of *ex vivo* cultured peripheral blood-derived EPCs and their effect on injured brain tissue after intravenous administration. To track the homing of implanted EPCs in injured brain tissues, EPCs were labeled with DAPI and BrdU *in vitro* before transplantation. EPCs were transplanted into the host animal through peripheral administration through the femoral vein, and homing of EPCs was evaluated. The integration of intravenously injected EPCs into the injured brain tissue was demonstrated. Immunohistochemical staining showed that microvessel density in the perifocal region of EPCs-transplanted rats was significantly increased, and the numbers of BrdU+ cells in the DG of subventricular zone were increased in EPCs-transplanted rats as compared to the control group. Transplanted EPCs may play an important role in reestablishing the endothelial integrity in the vessels after brain injury and further contribute to neurogenesis. EPCs enhanced recovery following brain injury in a rat model of TBI.

## Introduction

Endothelial progenitor cells (EPCs) are derived from a common hematopoietic precursor cell with a high proliferative potential, and have been generally thought to contribute to vasculogenesis and repair of endothelial cells (ECs)^1–3^. Circulating EPCs can differentiate into endothelial cells and then exert effects on angiogenesis. Angiogenesis plays a critical role in tissue repair, wound healing, tumor growth and stroke^4–6^. Injection of circulating EPCs into the hindlimb, lung, or myocardial ischemia of animals could result in incorporation of circulating EPCs into neovasculature at the site of ischemia, suggesting that circulating EPCs contribute to formation of new blood vessels in the adult tissue^7–9^. We had demonstrated a close correlation between an increase in circulating CD34+ cells in response to traumatic injury and angiogenesis in traumatic brain injury (TBI) rat brain^10^.

The exact roles of transplanted EPCs in reestablishing the endothelial integrity of vessels after brain injury and their therapeutic values in neural recovery remain unclear. In the current study, we tested the homing of *ex vivo* cultured EPCs and their effect on injured brain tissue after intravenous administration.

## Materials and Methods

All experimental procedures were approved by the Care of Experimental Animals Committee of Zhengzhou University and performed in accordance with the relevant guidelines and regulations.

### Isolation and culture conditions of endothelial progenitor cells (EPCs) from rat peripheral blood

Six to eight week-old male Wistar rats (weight: 220–300 g) were anesthetized via intraperitoneal injection with 1% of sodium phenobarbital (30 mg/kg). Peripheral blood was collected from the heart. Peripheral blood mononuclear cells (MNCs) were isolated by density gradient centrifugation using Ficoll-Paque PLUS (Herause, Germany). The mononuclear cell fraction was carded, washed and centrifuged at 1400 rpm for 10 minutes. The cells were suspended in EBM-2 medium (Clonetics, USA) supplemented with 10% fetal calf serum (FCS, Xiamen Tebao Bioengineering Company), vascular endothelial growth factor (VEGF), human fibroblast growth factor-B (hFGF-B), human epidermal growth factor (hEGF), and plated on rat-derived 10 μg/ml fibronectin-coated plates (Sigma Chemical). After culturing for three days, floating cells were removed by washing with potassium-buffered saline (PBS), and new media was added to the adherent cells. The cells were maintained in culture for seven days^2, 3^.

### Characterization of EPCs

After seven days in culture, MNCs were incubated with 2.4 µg/ml of DiI–labeled acetylated low-density lipoprotein (LDL (Biomeda,Shanghai) at 37 °C for 1 hour. The cells were washed three times with PBS and fixed with 2% PFA for 10 minutes. After washing with PBS, FITC-labeled lectin from ulexeuropaeus agglutinin (UEA) (Biomeda) was added to the cells for 1 h. Samples were washed with PBS, and viewed under an inverted fluorescent microscope and a laser scanning confocal microscope (Leica, Germany). Cells exhibiting double-positive fluorescence were identified as differentiating EPCs^2, 3^.

For characterization of endothelium markers, immunofluorescence was performed using rabbit polyclonal antibodies against CD31 and CD34 (Santa Cruz, USA). The cells were briefly washed three times with cold PBS and fixed with 2% PFA for 15 minutes. 3% bovine serum albumin (BSA) in PBS was then added for 30 min. Afterwards the cells were incubated at room temperature with CD31 and CD34 (1:500 dilution) with 3% BSA in PBS for 1 hour. After washing three times with PBS for 10 minutes, the cells were exposed to goat anti-rabbit Rhodamine (TRITC)-conjugated antibody (1:1000 dilution) for 1 hour in the dark. Nuclei were stained with Hoechst (4-,6-diamidino-2-phenylindole, Roche, Basel, Switzerland) for CD31 and CD34-positive EPCs. Negative control was incubated with PBS instead of primary antibody^2, 3^.

EPCs at 7^th^ day of culture were subjected to flow cytometric analysis. Cells were stained with FITC-conjugated CD34 (BD Biosciences,USA) and PE-conjugated stem cell marker CD133 (BD Biosciences) antibodies. Isotype-matched antibodies served as controls. After staining, the cells were fixed with 1% paraformaldehyde and analyzed by flow cytometry (Becton Dickinson, USA)^2, 3^.

### Labeling of EPCs

To track the homing of implanted EPCs in injured brain tissue, EPCs were labeled with green florescent protein (GFP) transfected by lentivirus (Genechem,China) and bromodeoxyuridine (BrdU) (Sigma Chemical) *in vitro* before transplantation after six days of culture. EPCs were briefly digested with 0.25% trypsin and re-suspended at 1 × 10^6^/ml in (DMEM) containing DAPI (10 µg/ml) for 20 minutes at 37 °C and then washed three times with PBS. GFP labeling was confirmed using an inverted fluorescent microscope. The EPCs were extensively washed by PBS and incubated with 10 mmol/ml BrdU in EC medium at 37 °C Twenty-four hours later, BrdU labeling was confirmed by BrdU staining. EPCs were then resuspended in 2 ml heparinized (10 units/ml) saline at 1 × 10^6^/ml for tail vein injection.

### Animal Model

A total of 48 adult male Wistar rats (weight: 300–350 g) were individually housed under a 12 hour light–dark cycle with regular food and water supply.

To test the homing of transplanted and labeled EPCs, TBIs were induced using a fluid percussion injury device (MODEL01-B, NEW SUN, CA, USA) in eight rats. The rats were briefly anesthetized with 2% halothane and then placed in a stereotatic frame. A 3.8 mm craniotomy was performed over the right parietal skull to expose the dura (4.0 mm posterior from bregma and 3.0 mm lateral to the sagittal suture). A luer-loc connector (2.6 mm inner diameter) was cemented to the skull with cranioplastic cement. A syringe filled with sterile saline was inserted into the luer-loc syringe fitting and connected to the fluid percussion device. The pressure pulse (200–233 kPa) and pulse duration were measured by a transducer attached to the fluid percussion device. Immediately after TBI, the incision was sutured and the rats were allowed to recover from anesthesia^11^.

To assess the effect of transplantation of EPCs on neurobehavior, 24 adult male Wistar rats were randomly assigned to the control (12 in each group) and EPCs-transplanted groups. TBI was induced using a fluid percussion injury device in 24 rats.

To assess the effect of transplantation of EPCs on the neurogenesis and angiogenesis after TBI, the other 16 rats (8 in each group) were randomly assigned to the control and EPC-transplanted groups. TBI was induced using a fluid percussion injury device in these rats. For analysis of neurogenesis, the EPC-transplanted rats were administered BrdU (75 mg/kg every 6 hour)) intraperitoneal1 day after the transplantation of unlabeled EPCs,and PBS was injected into the peritoneal cavity of the control group^12^. On the seventh day, 24 hours after the last BrdU injection, the rats were sacrificed and transcardially perfused with 0.1 mL cold PBS for 5 minutes followed by 4% cold paraformaldehyde for 17 minutes.

### Transplantation of *ex vivo* expanded EPCs

After the induction of TBI, rats were injected with DAPI and BrdU labeled EPCs (1 × 10^6^ cells/rat) to test the homing and unlabeled EPCs to assess their effect on the neurogenesis, angiogenesis and the neurobehavior by tail vein injection. Injection of EPCs was performed using microscopic evaluation to ascertain the complete venous administration into each host animal.

### Histological assessment of transplanted animals

One week after the procedure, the rats were perfused with heparinized saline and sacrificed to test the homing and the effect on the neurogenesis and angiogenesis The rat brains were embedded in Tissue-Tek OCT compound (Miles, Inc), frozen in 2-methylbutane (Fisher Scientific), and cooled on dry ice. Coronal brain sections (8 to 15 µm thick) were cut on a cryostat and thaw-mounted onto gelatin-coated slides. Some frozen sections were observed under an inverted fluorescent microscope to check the distribution of fluorescent-labeled EPCs by two independent observers who were blinded to the experimental conditions.

For BrdU immunostaining, DNA was first denatured by incubating the brain sections in 50% formamide 2X SSC at 65 °C for 2 hours and then in 2 N HCl at 37 °C for 30 minutes. Sections were treated with block endogenous peroxidase, incubated with a mouse monoclonal antibody against BrdU (1:1000 dilution) (Boehringer Mannheim) overnight followed by incubation with biotinylated secondary antibody (1:200 dilution) for 1 hour. Finally, the sections were developed with diaminobenzidine for 5 minutes. BrdU-labeled EPCs and neurogenesis were observed under a light microscope (Leica) by two independent observers who were blinded to the experimental conditions.

To assess the angiogenesis, CD34 immunostaining was performed. non-specific endogenous peroxidase activity was blocked by treating the sections with 3% hydrogen peroxide in methanol for 30 minutes. The antigen was recovered by boiling the sections in 10 mM citrate buffer (pH 6.0) for 10 minutes. Nonspecific binding was blocked with 1% non-immune serum in PBS for 30 min. The sections were then incubated with a monoclonal mouse anti-CD34 antibody (1:100 dilution) at 4 °C overnight. The sections were then washed with PBS, incubated with a biotinylated goat-anti-mouse secondary antibody (1:100 dilution) (Santa Cruz Biotechnology) for 2 hours at 37 °C, washed again, and incubated with an avidin peroxidase conjugate solution (1:100 dilution) (Santa Cruz Biotechnology, CA,USA) for 1 hour.Lastly, the sections were developed with diaminobenzidine for 5 minutes to generate a brown staining. The number of CD34^+^ endothelial-like cells in each section was counted in five fields with the highest densities by two independent observers who were blinded to the experimental conditions.

### Neurobehavioral tests

Modified Neurological Severity Score Test—The modified Neurological Severity Score (mNSS) test^13^ includes motor, sensory, reflex, and balance assessments, with the highest possible score being 18. A score of 13–18 indicates severe injury, 7–12 indicates moderate injury, and 1–6 indicates mild injury.The mNSS test was performed in the control and EPCs-treated groups (12 rats in each group) prior to and on the 1^st^, 3^rd^, 5^th^, 7^th^, 14^th^, 21^st^, and 25^th^ days after the TBI procedure.

### Statistical analysis

Data in the control and TBI groups were analyzed by a two-way ANOVA. All data are presented as mean ± SD. Values of p < 0.05 were considered to be statistically significant.

## Result

### Culture of EPCs

**Figure 1:**
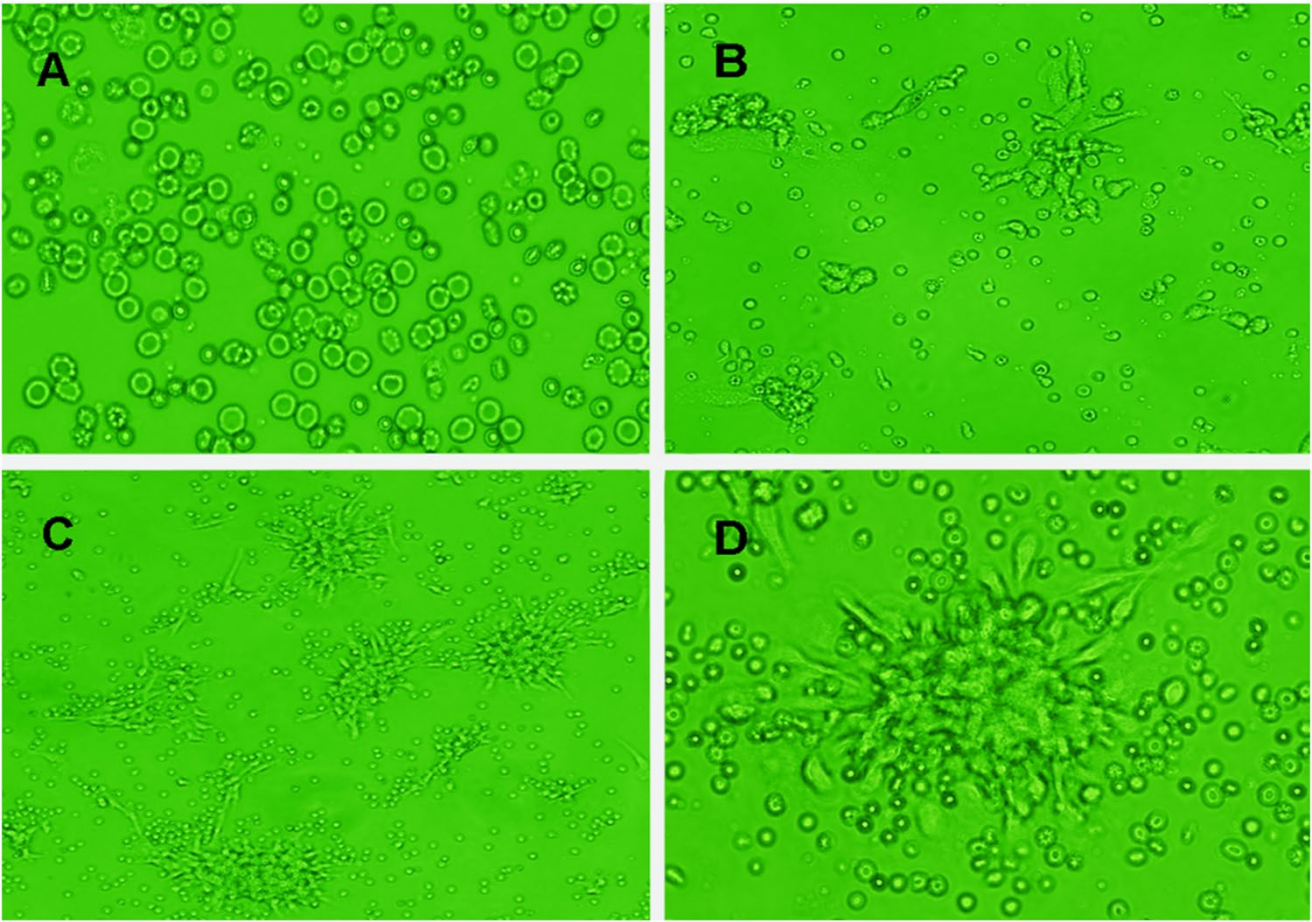
Rat peripheral blood-derived EPCs in culture. When peripheral blood mononuclear cells (MNCs) were cultured on fibronectin, attached cells (**A**) and typical morphology (spindle cell shape) of rat EPCs were observed after a few days of culture (**B**). Culture of MNCs resulted in the emergence of colonies with characteristic spindle-shaped EPCs. The colonies were well-formed after seven days of culture (**C**,**D**).

EPCs were expanded by culturing from MNCs isolated from peripheral blood. EPCs were expanded from attached cells (Fig. 1A) and cell clusters (Fig. 1B) isolated from culture of total MNCs. After culturing for seven days, the cells showed enhanced differentiation, such as cord-like structure (Fig. 1C) and typical EPC colony-forming units (Fig. 1D). EPCs were then characterized by the appearance of spindle-shaped cells projecting from a central cluster of round cells.

### Characterization of EPCs expanded *ex vivo*

**Figure 2:**
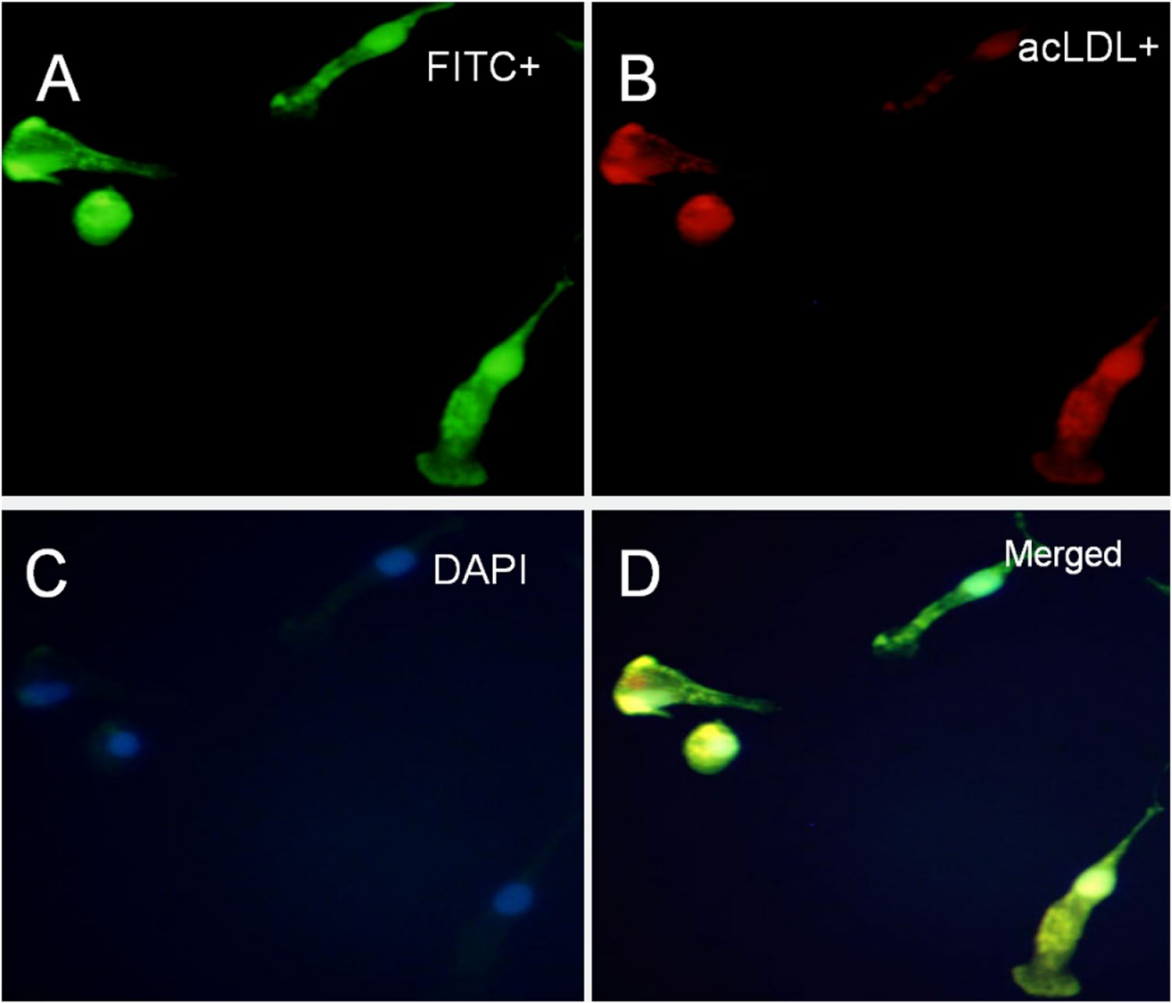
Identification of EPCs. After seven days of culture, EPCs were incubated with Dil-Ac-LDL and FITC-labeled lectin. Fixed cells showed the uptake of DiI-acetylated LDL and FITC-labeled lectin, and were double positive for DiI-Ac-LDL and FITC-lectin-UEA-1.

**Figure 3:**
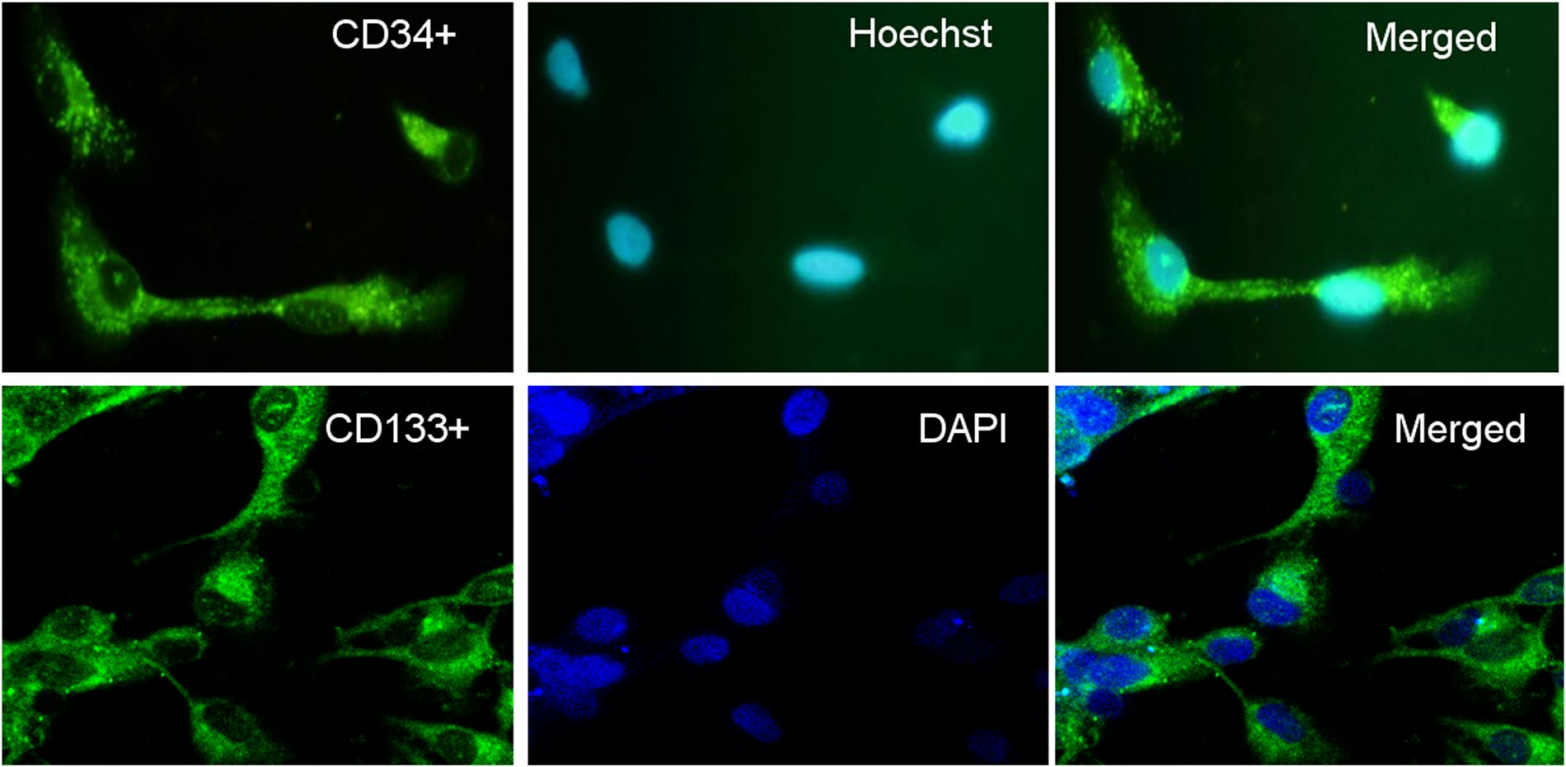
Identification of EPCs. After seven days of culture, the cells were labeled with fluorescent antibodies against CD34 or CD133, and positive staining was seen in the cultured EPCs.

The evaluation of characterization of EPCs was performed after seven-day culture of total MNCs. The adherent cells were double stained by Di-I-Ac-LDL and FITC-labeled lectin (Fig. 2A–D). These cells expressed endothelial cell-specific antigens (CD34 and CD31), which confirmed that the major population of adherent cells were EPCs (Fig. 3A–F). Expression of endothelial surface markers CD34 on EPCs were analyzed by flow cytometry, and 82.6% of cells were CD34-positive. EPCs showed higher expression of the stem cell marker CD133.

### Labeling of EPCs

**Figure 4:**
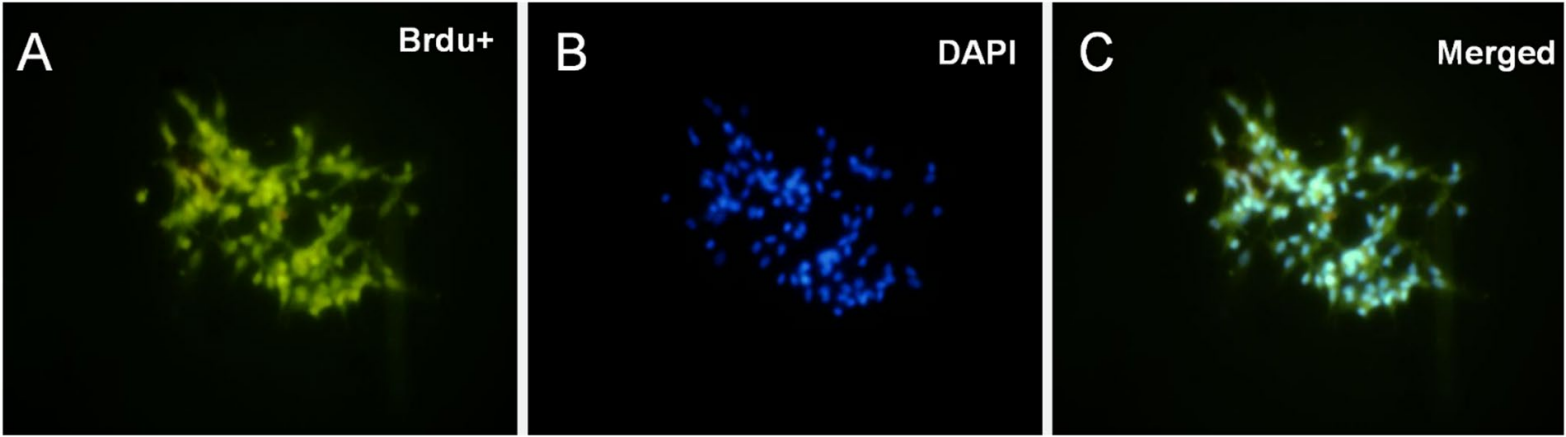
BrdU labeling of EPCs. BrdU-positive cells in culture before transplantation, and majority of the cultured EPCs were BrdU-positive.

Before transplantation, the cells were analyzed with BrdU labeling. Fig. 4A–C presents a series of photographs of BrdU-positive cells before transplantation. Incubation of cultured cells with BrdU revealed that >70% (five fields randomly) EPCs showed uptake of BrdU over a 24-hour period. DAPI-labeled EPCs were seen under a fluorescence microscope (Fig. 4B).

### Incorporation of EPCs into the injured brain

**Figure 5:**
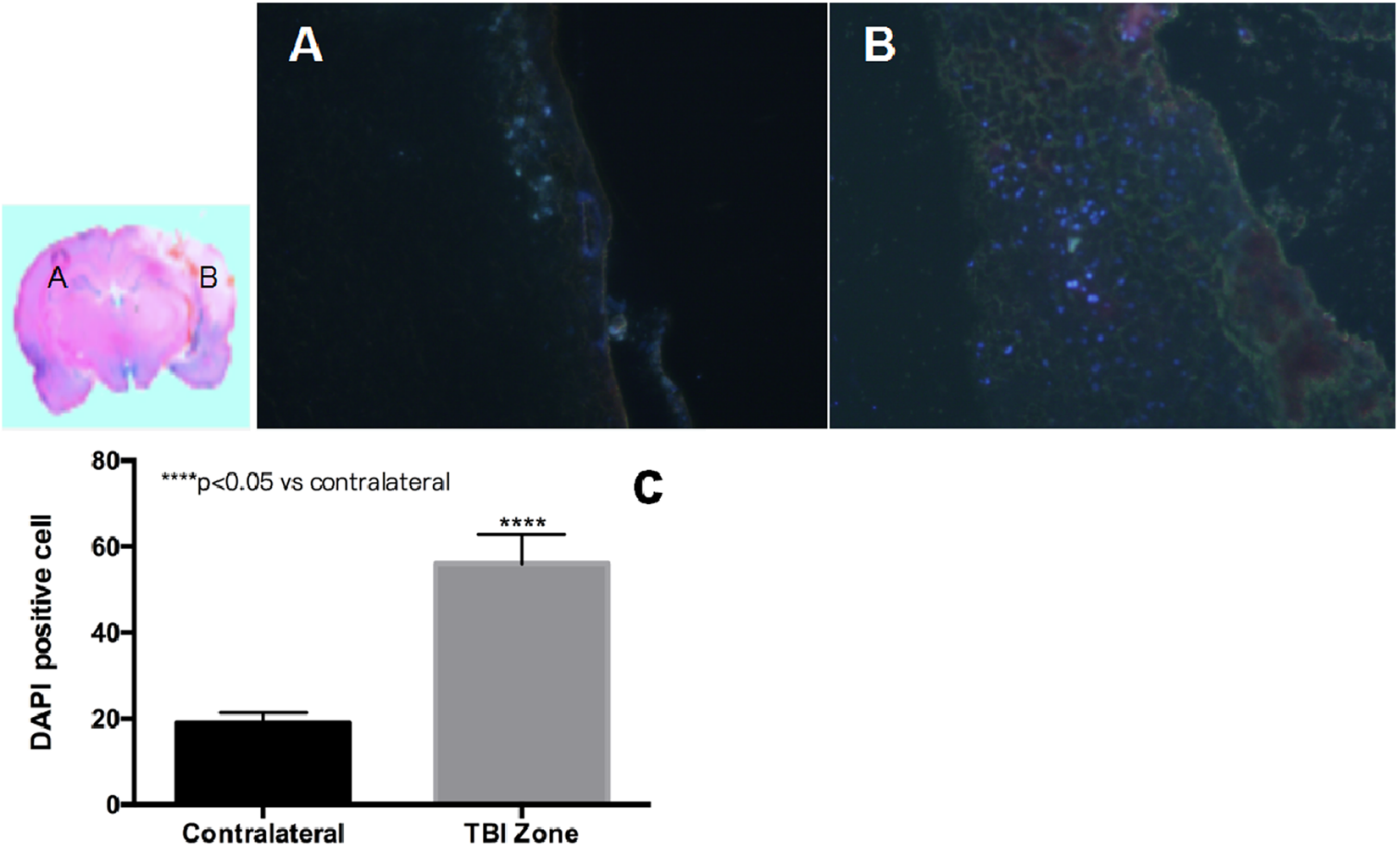
Distribution of DAPI-positive EPCs. Seven days after tail vein transplantation of EPCs, several DAPI-positive EPCs were detected in the TBI zone, and few DAPI-positive EPCs were detected in the contralateral hemisphere. DAPI-positive cells were significantly higher in the ipsilateral hemisphere as compared to the contralateral hemisphere (P < 0.05).

DAPI-labeled EPCs were intravenously administered 50–120 minutes after TBI. Specific immunofluorescence for DAPI was found in the TBI brain zone in all TBI animals (n = 9), while few transplanted EPCs were found in the contralateral hemisphere. We were able to detect injected EPCs at about 45 ± 12.6 cells per 200X microscopic field (Fig. 5).

**Figure 6:**
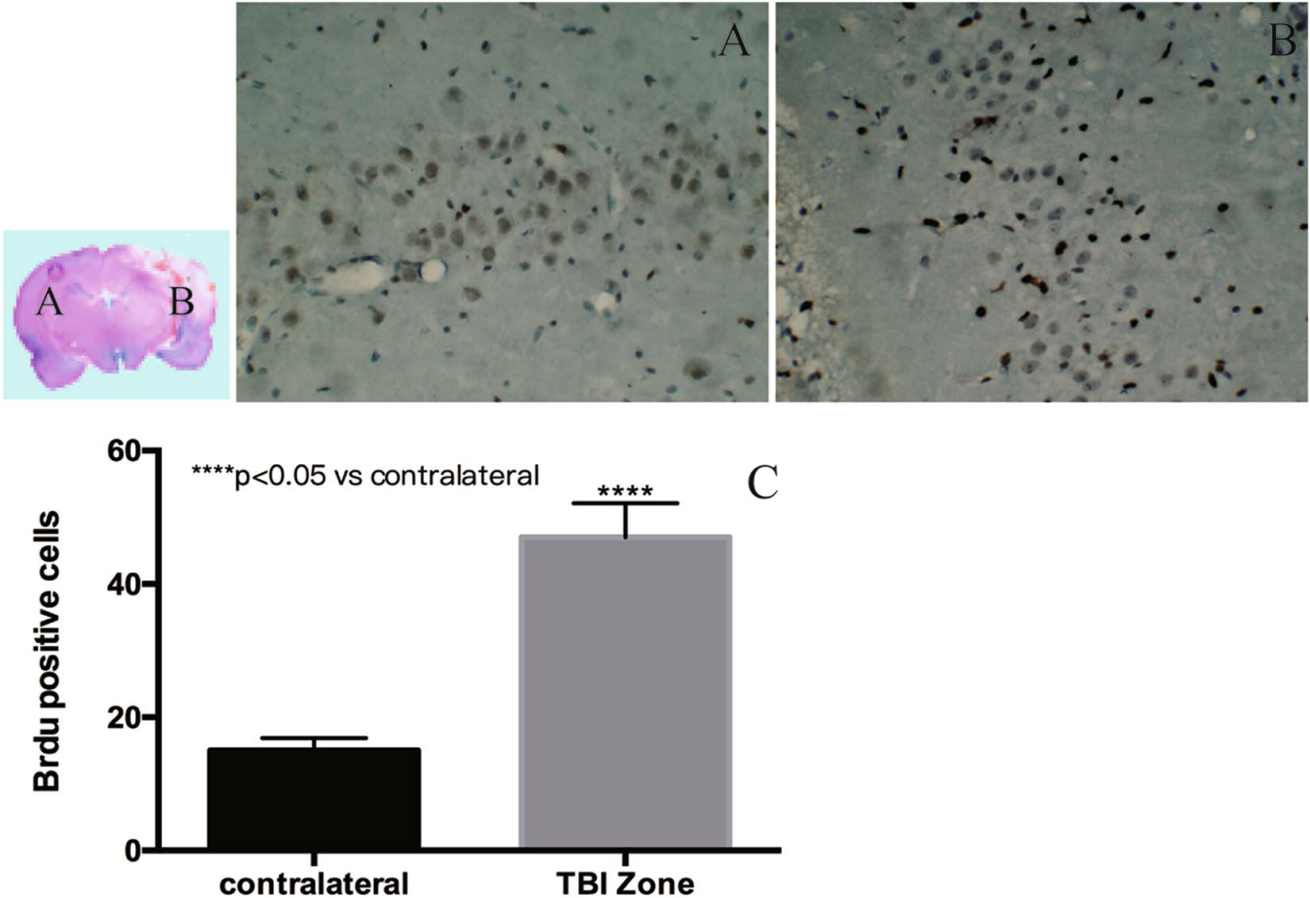
Distribution of BrdU-positive EPCs. Seven days after tail vein transplantation of EPCs, several BrdU-positive EPCs were detected in the TBI zone, and few BrdU-positive EPCs were detected in the contralateral hemisphere. BrdU-positive cells were significantly higher in the ipsilateral hemisphere as compared to the contralateral hemisphere (P < 0.05).

BrdU-immunoreactive cells were present in the TBI brain zone seven days after TBI. Few BrdU-immunoreactive cells were detected in the contralateral hemisphere (Fig. 6).

### Effect of EPCs on hippocampal neurogenesis

**Figure 7:**
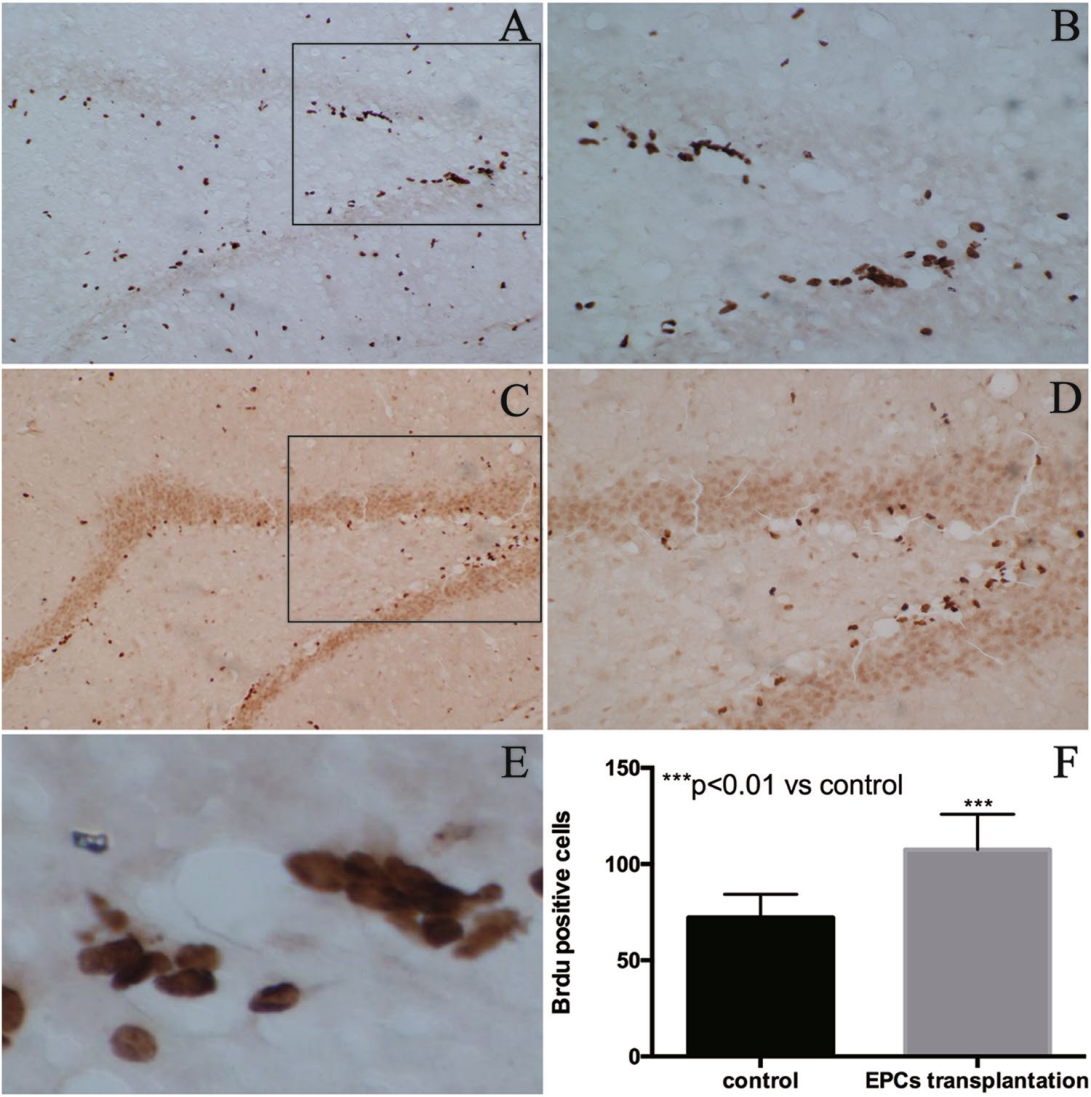
Neurogenesis in the DG of adult hippocampus after EPC transplantation. BrdU+ cells expression in the DG of the hippocampus of the EPCs transplanted group (**A**,**B**) and the control group (**C**,**D**). The BrdU-positive cells in the hippocampus were in clusters (**E**). The number of BrdU-positive cells in the adult hippocampus increased after EPC transplantation as compared to the control (P < 0.05 versus the control group, 100×). Data are presented as means ± SD (**F**).

To evaluate the effect of EPCs on neurogenesis in the dentate gyrus (DG) of hippocampus, the rats of the control and transplanted groups were sacrificed on the seventh day at 24 hr after BrdU injection. The BrdU-positive cells in the hippocampus were found in clusters (Fig. 7E). The number of BrdU-positive cells in the EPCs transplanted group was higher than the control group (Fig. 7A–D). There was an apparent difference between the control and EPC transplanted groups in the number of BrdU-positive cells in the DG of hippocampus (Fig. 7F). These results showed that transplanted EPCs increased the number of BrdU-positive cells in the hippocampus and promoted neurogenesis.

### Effect of EPCs on angiogenesis

**Figure 8:**
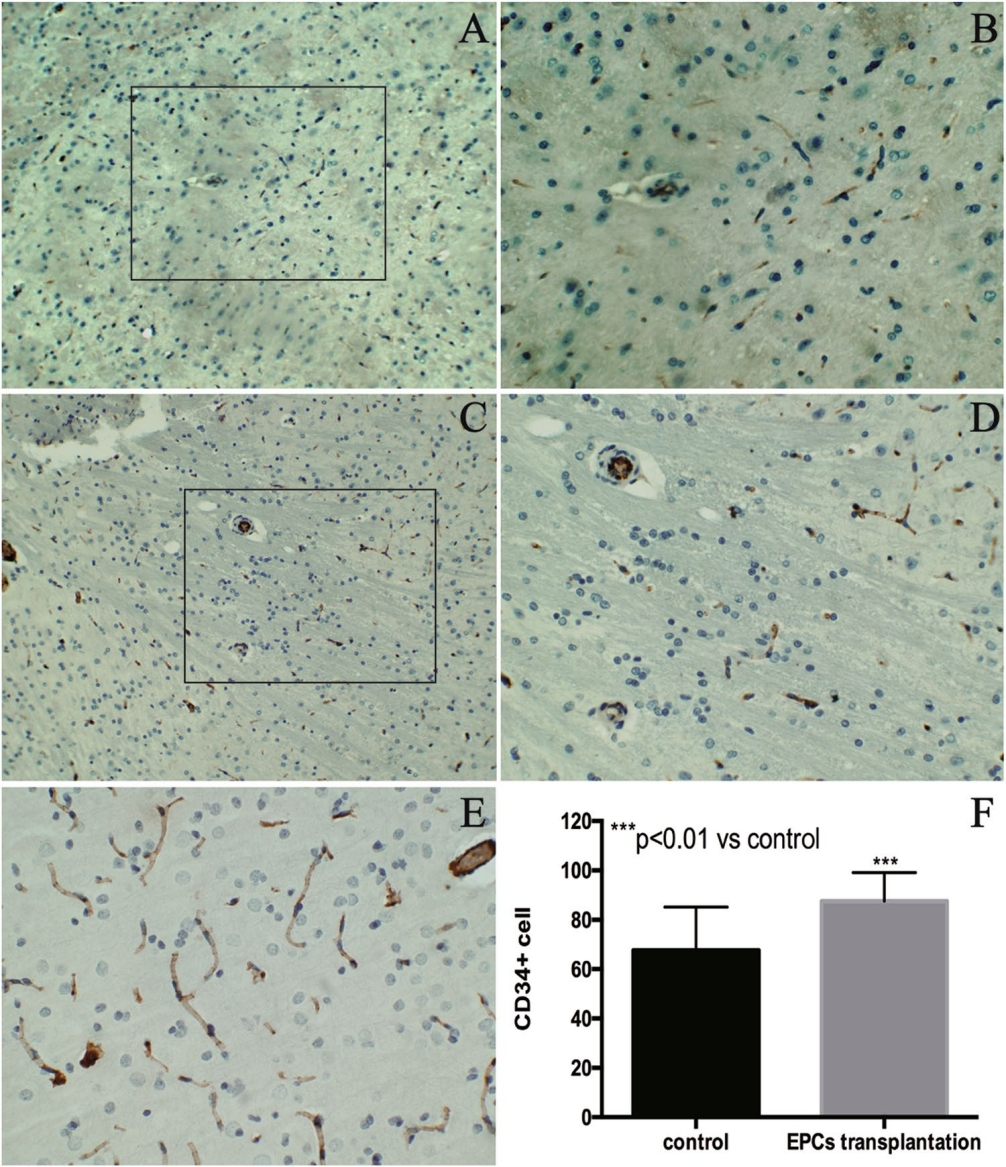
Effect of EPCs on angiogenesis. CD34 was used as a marker of microvascular endothelial cells to evaluate the effect of EPCs on angiogenesis. CD34+ cells expression in the EPCs transplanted group (**A**,**B**) and the control group (**C**,**D**). CD34+ endothelial–like vessel-lumen structure appeared in the brain tissue (**E**). The number of CD34+ cells in the traumatic zone increased after EPC transplantation as compared to the control group (P < 0.05 versus the control group, 100×). Data are presented as means ± SD (**F**).

This study utilized CD34 as a marker of microvascular endothelial cells to evaluate the effect of EPCs on angiogenesis. The number of endothelial-like cells was analyzed in the ipsilateral traumatic zone seven days after EPC transplantation. CD34+ cells in the transplanted group were higher than the control group (Fig. 8A–D). CD34+ endothelial–like vessel-lumen structure appeared in the brain tissue (Fig. 8E). CD34+ cells were significantly increased after EPC transplantation as compared to the control group (Fig. 8F). These results showed that transplanted EPCs increased the number of CD34+ cells and promoted the angiogenesis.

### Neurobehavioral tests

**Figure 9:**
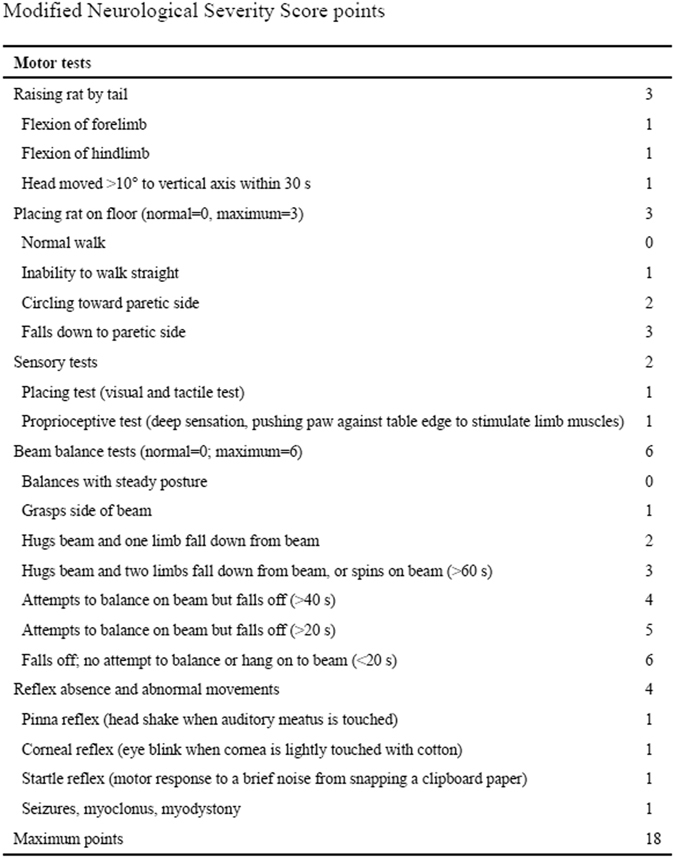
Modified Neurological Severity Score points.

**Figure 10:**
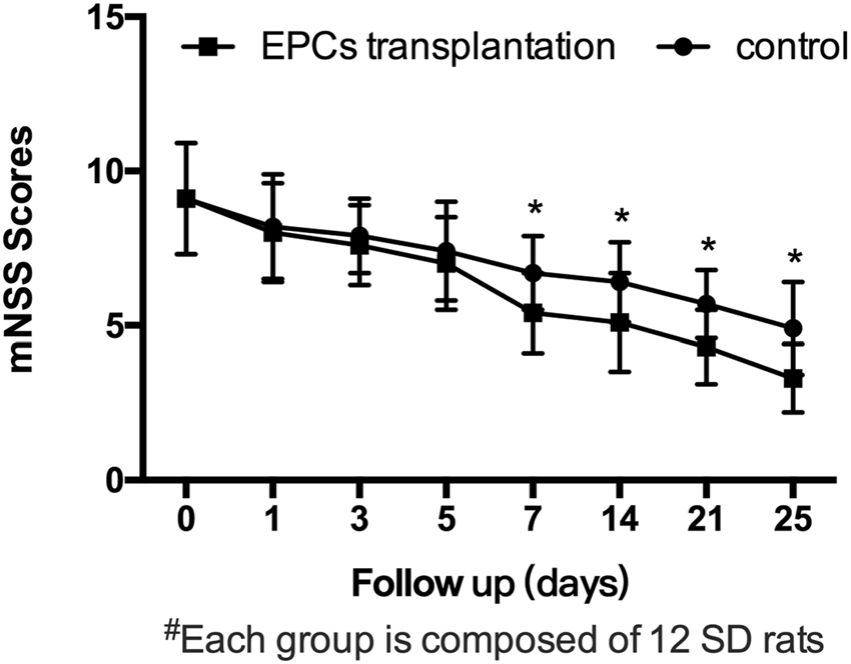
Neurobehavioral tests. The mNSS test was performed in the control and EPCs-treated groups (12 rats in each group). Neurological function was evaluated by mNSS prior to and 1^st^, 3^rd^, 5^th^, 7^th^, 14^th^, 21^st^, and 25^th^ days in the control and EPC-transplanted rats after TBI. Quantification of mNSS test results showed that the EPC treatment improved neurofunctional outcomes. *P < 0.05 versus the control group. Data are presented as means ± SD.

The mNSS test (Fig. 9) was performed in the control and EPCs-treated groups (12 rats in each group). The mNSS tests were used to determine whether EPC treatment improved the neurological function of TBI rats. The motor, sensory, and beam balance assessments of the mNSS test were conducted prior to and on the 1^st^, 3^rd^, 5^th^, 7^th^, 14^th^, 21^st^, and 25^th^ days following the TBI procedure in the control and EPC-transplanted rats. There were no differences between the groups 1^st^, 3^rd^ and 5^th^ days after the TBI procedure, but the mNSS scores of the EPC-transplanted group were lower than those of the control group on day 7 (5.4 ± 1.3 versus 6.7 ± 1.2, respectively; P < 0.05),14 (5.1 ± 1.6 versus 6.4 ± 1.3, respectively; P < 0.05), 21 (4.3 ± 1.2 versus 5.7 ± 1.1, respectively; P < 0.05) and 25 (3.3 ± 1.1 versus 4.9 ± 1.5, respectively; P < 0.01) after the TBI procedure (Fig. 10).

## Discussion

Endothelial progenitor cells (EPCs) have demonstrated therapeutic potential in various diseases^14–16^. The main finding of this study was that *ex vivo* cultured EPCs selectively tracked to injured brain tissue but not to normal tissue after traumatic brain injury by peripheral administration. EPCs may play an important role in reestablishing the endothelial integrity of vessels after brain injury, and further contribute to neurogenesis. In this study, the neurovascular protective effect of EPC treatment after brain injury was demonstrated. EPCs promoted focal angiogenesis and neurogenesis.

The homing or recruitment of circulating EPCs into injury or ischemic sites is an important process for executing their angiogenic and repair functions^17^. EPCs can home to areas of tissue injury, thus contributing to endothelial regeneration and neovascularization. Homing of EPCs to injured brain tissue indicates a potential repair mechanism. The capillaries may be destroyed, and ECs may undergo apoptosis or necrosis. It is therefore conceivable that EPCs can travel to these capillaries where the adult EC phenotype is lost^16, 18^. This hypothesis is supported by the findings that apoptotic mature ECs are replaced by novel functional cells. A pilot study and randomized controlled trial in patients treated with autologous transplanted bone-marrow cells in ischemic limb muscles showed a sustained significant effect of this therapeutic angiogenesis^19^. Notably, EPCs were able to improve the overall function in ischemic tissues by increasing neovascularization and attenuating organ damage^3, 20, 21^.

Angiogenesis plays a critical role in tissue repair, wound healing, tumor growth and stroke^4–6^. The contribution of EPCs in angiogenesis has been documented in the recovery processes of various diseases, such as myocardial ischemia, limb ischemia, and ischemic stroke^22, 23^. In the present study, vascular density was higher in the EPCs transplanted group than in the normal control group, suggesting that transplanted EPCs promoted angiogenesis after brain injury. New vessels may form due to the proliferation and migration of circulating EPCs^24^. The present study indicated that *ex vivo* culture-expanded EPCs home to injured brain tissue after transplantation, and may be used to successfully promote neovascularization of injured tissues.

Previous studies have shown that angiogenesis is coupled with neuroprotection and neurogenesis^22, 25^. Systemic administration of human cord blood-derived CD34-positive cells in mice after 48 hours of ischemia-induced neovascularization in the ischemic zone, promoted neuronal progenitor cell migration and survival, and thereby improved cortical expansion 90 days after cell transplantation^26^. Transplanted EPCs may play an important role in neurogenesis after brain injury and further contribute to neural recovery. In the present study, neurogenesis was higher in the EPCs transplanted group than in the normal control group, suggesting that the transplanted EPCs promoted neurogenesis after brain injury.

Endothelial progenitor cells have demonstrated therapeutic potential in various diseases. Injection of circulating EPCs into the hindlimb, lung, or myocardial ischemia of animals have been shown to promote functional recovery^14–16^. Angiogenesis plays a critical role in tissue repair, wound healing, tumor growth and stroke^4–6^. In the present study, neurologic testing showed transplanted EPCs improved functional recovery in animal models of TBI. Transplanted EPCs may contribute to formation of new blood vessels and neurogenesis to promote functional recovery.

## Conclusion

Our data demonstrated the successful integration of intravenously administered *ex*-*vivo* expanded EPCs in the injured brain tissue and the neuro-vascular protective effect of EPCs after traumatic brain injury in an animal model. With extremely low EPCs in peripheral blood, *ex*-*vivo* EPCs transplantation may be a promising method and therapeutic option for neurogenesis and functional recovery promotion in patients with traumatic brain injury.
